# Brief Tale of a Bacteraemia by *Rhodococcus equi*, With Concomitant Lung Mass: What Came First, the Chicken or The Egg?

**DOI:** 10.4084/MJHID.2011.006

**Published:** 2011-01-17

**Authors:** Vincenzo Savini, Prassede Salutari, Marco Sborgia, Iole Mancini, Gioviana Masciarelli, Chiara Catavitello, Daniela Astolfi, Claudio D’Amario, Giuseppe Fioritoni, Antonio Spadaro, Domenico D’Antonio

**Affiliations:** 1 Clinical Microbiology and Virology, Department of Transfusion Medicine, Spirito Santo Hospital, Pescara (Pe), Italy; 2 Department of Haematology, Spirito Santo Hospital, Pescara (Pe), Italy; 3 Department of Radiology and Biomedical Imaging, Spirito Santo Hospital, Pescara (Pe), Italy; 4 Clinical Pathology, San Liberatore Hospital, Atri (Te), Italy

## Abstract

*Rhodococcus equi* is an uncommon Gram positive, variably acid-fast pathogen, that appears as hard to treat mostly owing to the establishment of intracellular niches. Lack of interpretive criteria for susceptibility testing may lead to under-reporting or overestimation of resistances, whereas knowledge about this pathogen’s clinical impact may be affected by erroneous phenotype-based characterization at a genus and species level.

We present the case of a bacteraemia with a concomitant lung mass in a lymphoma patient, that further highlights the emergence of rhodococcal diseases as a matter for concern in the fields of infectious diseases and haematology.

## Introduction

*Rhodococcus equi* is a zoonotic organism that has been collected from soil, water, horses, cattle, swines and wild birds; although it is found in stools of many grazing animals, foals show a unique susceptibility to clinical disease. They usually present with a subacute to chronic purulent bronchopneumonia, complicating with abscess formation and suppurative involvment of distant sites including colon, lymph nodes and skin. The organism rarely affects other herbivores, but may cause swine granulomatous submaxillary and submandibolary lymphadenitis. In humans, it is responsible for rare, potentially fatal diseases, predominantly occurring in compromised patients with neutropenia, underlying HIV infection, lymphoma, T-cell prolymphocytic leukaemia, chronic and acute myeloid/chronic and acute lymphoblastic leukemia, chronic renal failure, end stage liver disease, lung cancer, alcoholism, diabetes mellitus, along with those undergoing splenectomy, or receiving corticosteroids and/or immunosuppressive drugs (i.e. for rheumatoid arthritis, sarcoidosis, systemic lupus erythematosus and antineutrophil cytoplasmic antibodies positive vasculitis), solid organ transplantation (kidney, pancreas, kidney-pancreas, heart, liver, lung) and HSCT (Hematopoietic Stem Cell Transplantation). Conversely, only 10–15% of all cases happens in the apparent absence of any immune system impairment. Mortality of about 11%, 50–55%, and 20–25% is observed in immunocompetent people, AIDS (Acquired Immune Deficiency Syndrome) patients, and compromised hosts without HIV infection, respectively.^1^–^8^

## Case report

A 54-year-old male patient suffering from refractory non-Hodgkin lymphoma with peripheral B lymphocytes, LLC type (diagnosed 6 years before), was admitted to the Haematology department of the Pescara Civic Hospital due to the onset of fever (up to 39°C). Blood samples (two bioMérieux BacT/Alert aerobe bottles plus one for anaerobe organisms) were taken for culture; a second set (for only aerobes) was sent to laboratory 30 min later, then meropenem was started, without success.

After 48 h incubation, the aerobe samples were detected as positive by the instrument. Smear staining showed Gram positive coccobacilli, with cultures growing *R. equi*-like colonies (see below) on sheep blood agar (> 200 colony-forming units [CFU]/plate), after 72 h incubation, in ambient air, at 37^o^C. BioMérieux Vitek2 identified the isolate as *Kocuria kristinae* (excellent identification, 99% certainty); anyway, *Kocuria* species are known to only include cocci, not coccobacilli; so, 16S RNA sequencing was performed and finally confirmed the isolate as *R. equi*, as expected.^8^,^9^

A Clinical and Laboratory Standards Institute (CLSI) disc diffusion assay^10^ was performed, with the isolate showing resistance to penicillin, oxacillin, clindamycin and tetracycline, and susceptibility to all among erythromycin, ciprofloxacin, cotrimoxazole, rifampin, teicoplanin and vancomycin. Notably, due to the lack of specific inhibition zone diameter breakpoints, interpretive criteria for staphylococci were used. Again, being resistance mechanisms still unclear, the strain was prudently considered as sharing PBP (Penicillin Binding Protein) mutation with oxacillin-resistant staphylococci, then pan-β-lactam resistant. Meropenem was then replaced by vancomycin (10-day administration), which led to resolution of fever within 48 h. Blood cultures performed after 1 week from starting glycopeptide did not grow any organisms.

**Figure 1:**
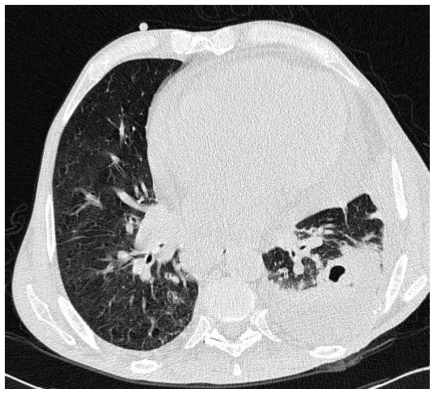
Patient’s lung nodule at CT scan.

The patient did not have any central venous lines, and was receiving no chemotherapies at the time that the bacteraemic episode was diagnosed. Of interest, stool and sputum cultures grew *R. equi*, both concurrently with bacteraemia and after the patient recovered from the latter; this was perhaps due to the intracellular survival of the organism, so that vancomycin could not eradicate colonization. Notably, a cavitated left upper lobe lung nodule (Figure 1) was observed at CT (Computed Tomography) scan and surgically removed, after resolution of the bloodstream infection. The omogenated tissue grew *R. equi* as a pure culture (>10^5^ CFU/ml), with all among blood, lung, stool and sputum isolates sharing the same behaviour towards drugs (genome comparison was not performed, unfortunately). Then, we wondered whether the organism moved from lung to blood, or vice versa. Finally, in spite of the well known diffusion of *R. equi* in livestock, the patient did not refer any contact with animals, so that the source of colonization remained unknown.

## Discussion

**Figure 2:**
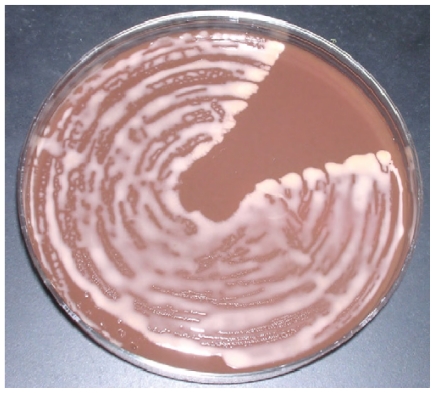
Colonies on chocolate agar plate (image taken from Yamshchikov AV et al. Lancet Infect Dis 2010; 10: 350–359, With the permission of the author).

Rhodococci are Gram positive organisms belonging to the group of mycolata (mycolic acid containing bacteria), that also incorporates the genera *Nocardia*, *Gordonia*, *Corynebacterium*, *Tsukamurella*, *Dietzia*, *Williamsia*, *Turicella*, *Skermania*, and *Mycobacterium*.^11^ The name *Rhodococcus* was firstly used by the German botanist Wilhelm Friedrich Zopf in 1891, when classifying pigment-producing bacterial and fungal microorganisms. The genus was then redefined in 1977 to include members of the rhodochrous complex, which contained nocardioform and mycobacterial-like species. *R. equi* (previously *Corynebacterium equi*) appears as an encapsulated, non-motile, catalase-positive, CAMP test-positive, strictly aerobe, pleomorphic coccobacillus (cells can be erroneously labeled as belonging to corynebacteria and micrococci), although rhodococci may be observed as cocci on solid media and tissues, but appear as pleomorphic (with long rods or filaments, mycelial branching and rudimentary beading) in liquid cultures. *R. equi* grows optimally at 30^o^C, forming large, mucoid, irregular, salmon-pink to red colonies after 2–7 days of incubation (Figure 2). Phenotype-based misidentification as *Corynebacterium* sp. has been obtained by using automated methods for typing. Finally, acid-fast staining cannot discriminate the organism from mycobacteria.^7^,^9^,^12^–^15^

*R. equi* was firstly isolated from foals with pyogranulomatous pneumonia in 1923, with the first human infection being diagnosed in 1967; major clinical manifestations include lung, liver, kidney, psoas muscle, subcutaneous, pelvic and brain abscesses, pneumonia (even with necrotizing cavitary lesions, pulmonary malakoplakia, mediastinal lymphadenitis, effusion/empyema, concomitant bacteraemia and disseminated infection), cutaneous wound infections, gastrointestinal ulcerations, diarrhea, meningitis, pericarditis, endocarditis, osteomyelitis, peritonitis (associated with ambulatory peritoneal dialysis) and peritoneal shunt infection, cervical adenopathy, lymphangitis, mastoiditis, endophtalmitis and sepsis. Clinical picture of low airway disease usually includes cachexia, fatigue and weight loss; these symptoms, along with the occasional and variable acid fastness of the pathogen and its predilection for causing upper-lobe cavitation, frequently lead to misdiagnosis as pulmonary tuberculosis. Due to survival inside histiocytes, diseases are commonly chronic and recurrent, with relapses occurring after brief courses of antibiotics, or during treatment.^5^–^8^,^12^,^13^,^16^,^17^

**Figure 3:**
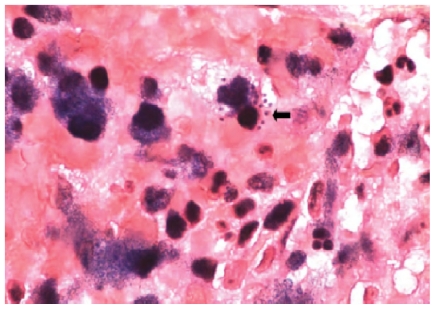
Lung tissue with intracellular coccobacillary forms (image taken from Yamshchikov AV et al. Lancet Infect Dis 2010; 10: 350–359, With the permission of the author).

In this context, malakoplakia is a dense infiltration of foamy histiocytes with intracellular coccobacilli and scattered concentric basophilic inclusions called Michaelis-Gutmann bodies, which likely represent infected macrophages with ingested bacteria; although not specific to *R. equi* infection (*Escherichia coli*, *Pasteurella multocida* and *Mycobacterium tuberculosis* may cause similar pathological changes), it is frequently associated with lung illnesses by this species, in compromised hosts (Figure 3).^7^ The surface soil of 50–95% of horse farms shows high concentrations of the microorganism, with inhalation of infected dust particles and aerosols appearing as the main route of transmission to foals. Also, these can acquire the infection through direct inoculation of wounds and mucous membranes, as well as by metastatic dissemination to distant sites. Similarly, humans may fall ill after ingestion, inhalation or direct inoculation of bacteria into skin lesions. Man disease is in fact strongly related to livestock exposure or farming environments, although apparent contact with animals may be lacking and no clear evidence of endogenous colonization as a source for human illness exists so far. Finally, even if nosocomial and person-to-person transmission is rare, pneumonia in patients sharing the same hospital room has been described.^7^,^8^,^12^,^13^,^16^,^17^

Little is still known about this pathogen’s resistance to drugs; *in vitro* sensitivity to erythromycin, fluoroquinolones, rifampin, aminoglycosides, imipenem, glycopeptides, as well as penicillin, erythromycin, vancomycin, rifampin and minocycline resistance, have been observed over the years. Also, its behaviour under exposure to tetracycline, clindamycin and cotrimoxazole may vary. Resistance is presumed to occur owing to PBP mutation, increased antibiotic degradation, and β-lactamase production, but further studies are evoked to shed some light on this obscure field. Intrinsic along with acquired resistance to penicillins and cephalosporins (including loss of sensitivity during therapy) have been described, so that the use of β-lactams is controversial and combination with intracellularly active compounds (such as rifampin, erythromycin, azithromycin, and ciprofloxacin) should be preferred (since intrahistiocytic survival remains a major virulence trait). Again, the use of linezolid, as well as that of β-lactam/β-lactamase inhibitor combination has been proposed. Treatment length should be based on frequent radiographic as well as clinical assessment, with prolonged (9–12 months) therapy being recommended in immunosuppressed hosts; instead, a 2- to 8-week course of two antibiotics seems to be enough for localised diseases (such as mild-moderate pneumonia and soft tissue abscess). In addition, some published works suggest that a 2- to 6-weeks induction with an intravenous compound (i.e. vancomycin, or a carbapenem, or an aminoglycoside) is needed in compromised patients, then combined with up to 6 months of oral azithromycin or rifampin. The infection can be faced by antibiotics alone, although surgical treatment may be beneficial for debulking extensive disease.^4^,^7^,^8^,^13^,^16^,^18^,^19^

This brief communication may add to the current knowledge about bloodstream and respiratory infections by rhodococci; also, it firstly reports misidentification of *R. equi* as *Kocuria* sp., thus emphasizing the risk of wrongly typing bacteria, unless genome-based methods are used. Again, we would suggest microbiologists to prolong cultures (not only those for fungi) up to ≥48 h, aiming to observe slowly growing bacteria that would be otherwise missed; but above all, we would increase clinicians’ awareness of this pathogen’s morbidity in compromised people. 13 In this context, we believe a deeper attention shoud be given to uncommon nosocomial agents of infections, to establish specific criteria for sensitivity testing, to investigate among their mechanisms of resistance, to better understand the epidemiology in the environment and within hospitals, and to increase the knowledge and consciousness of their clinical impact and pathogenic traits, that will otherwise remain fragmentary and incomplete.
